# The Relationship Between Hepcidin and the Severity of Obstructive Sleep Apnea Syndrome and Obesity

**DOI:** 10.3390/life16040629

**Published:** 2026-04-08

**Authors:** Hikmet Coban, Emine Ayan, Mustafa Colak, Nurhan Sarioglu, Gulcin Yilmaz Gunes, Fuat Erel, Merve Yumrukuz Senel, Merve Akış Yılmaz

**Affiliations:** 1 Department of Pulmonology, Faculty of Medicine, Balikesir University, 10145 Balikesir, Türkiye; dremineayan@gmail.com (E.A.); mustafa.colak@balikesir.edu.tr (M.C.); nurhangencer@hotmail.com (N.S.); drgulcinyilmaz@gmail.com (G.Y.G.); fuat.erel@balikesir.edu.tr (F.E.); merve.senel@balikesir.edu.tr (M.Y.S.); 2 Department of Medical Biochemistry, Faculty of Medicine, Balikesir University, 10145 Balikesir, Türkiye; merve.akis@balikesir.edu.tr

**Keywords:** obstructive sleep apnea, hepcidin, obesity

## Abstract

The effects of chronic intermittent hypoxia associated with obstructive sleep apnea (OSA) on iron metabolism remain incompletely understood. This study aimed to evaluate whether serum hepcidin levels are associated with OSA severity independently of obesity and systemic inflammation. A total of 136 patients who underwent polysomnography for suspected OSA between April and December 2025 were included in the study. Participants were classified into control and OSA groups according to the Apnea–Hypopnea Index (AHI), and the OSA group was further categorized as mild, moderate, or severe. Demographic and anthropometric characteristics and Epworth Sleepiness Scale scores were recorded. Serum hepcidin levels were measured using an ELISA method and compared between groups, and their associations with clinical and polysomnographic parameters were analyzed. Serum hepcidin levels were significantly higher in patients with OSA than in the control group (48.83 ± 11.92 vs. 41.53 ± 12.43 ng/mL; *p* < 0.001) and increased progressively with disease severity. Hepcidin levels were not significantly correlated with conventional iron parameters but showed a strong positive association with the Oxygen Desaturation Index (ODI). In multivariable regression analysis, the positive relationship between AHI and serum hepcidin levels remained significant after adjustment for body mass index and C-reactive protein levels (*p* < 0.001). These findings suggest that elevated serum hepcidin levels in OSA are linked to hypoxic stress independently of obesity and systemic inflammation, indicating that hepcidin may represent a potential biomarker reflecting disease severity in OSA.

## 1. Introduction

Obstructive sleep apnea (OSA) is a common sleep disorder characterized by intermittent hypoxia, sleep disruptions, and increased sympathetic activity resulting from recurrent collapse of the upper airway during sleep. Male gender and obesity, along with advanced age, craniofacial structure, genetic predisposition, smoking, and alcohol use are the main risk factors for OSA [1]. Beyond these anatomical and demographic risk factors, intermittent hypoxia, the hallmark feature of OSA, is the primary determinant in the development of systemic pathological changes.

The hypoxia-reoxygenation cycles accompanying OSA trigger oxidative stress mechanisms, leading to the development of chronic inflammation [2]. Various studies have shown an increase in the levels of pro-inflammatory cytokines such as IL-6, TNF-α, and IL-1β during this process. C-reactive protein (CRP) levels, a sensitive indicator of systemic inflammation, are also known to be elevated in OSA patients compared to healthy adults. This inflammatory activation is thought to play a role in the development of metabolic, cardiovascular, and hematological complications associated with OSA [3].

To complement this hypoxia-induced inflammation, increased fat tissue accumulation around the upper airway and in the central region due to obesity causes a decrease in airway patency and an increased tendency for collapse during sleep [4]. However, obesity does not merely cause mechanical effects; it also leads to the development of chronic inflammation through adipocyte hypertrophy and cellular damage caused by hypoxia in adipose tissue. This dysfunctional adipose tissue is characterized by increased release of pro- inflammatory cytokines such as TNF-α, IL-6, IL-8, and MCP-1 [5]. The combined effect of the inflammatory response associated with intermittent hypoxia in OSA and the inflammatory processes accompanying obesity leads to further deepening of systemic inflammation [6].

Another critical yet often overlooked determinant in this inflammatory milieu is hepcidin, a liver-derived peptide hormone that serves as the principal regulator of systemic iron homeostasis. Hepcidin regulation is primarily governed by iron store status, erythropoietic activity, and the inflammatory response, while inflammation and iron overload increase its expression. By inhibiting duodenal iron absorption and the release of iron from macrophages and hepatocytes into the plasma, hepcidin limits the amount of iron entering the circulation. Particularly in infectious and inflammatory conditions, increased interleukin-6 (IL-6)-mediated hepcidin expression leads to iron sequestration within macrophages and a reduction in plasma free iron levels [7].

It is thought that the increased erythropoietic stimulation associated with chronic and intermittent hypoxia in OSA may modulate hepcidin levels via erythropoietin and that this mechanism may contribute to changes in iron metabolism [7]. However, the effect of hypoxia on hepcidin may vary depending on the type of hypoxia. Persistent hypoxia may suppress hepcidin expression via Hypoxia- Inducible Factor-1 alpha (HIF-1α) activation [8], while studies have shown that intermittent hypoxia strongly enhances peroxide-mediated hepcidin induction via the STAT3 signaling pathway [9].

Despite these physiological links, the clinical evidence regarding hepcidin levels in OSA remains conflicting. A major limitation of the existing literature is the lack of rigorous control for confounding factors such as obesity and systemic inflammation, both of which are themselves strong regulators of hepcidin. It therefore remains unclear whether the observed alterations in hepcidin levels are a specific consequence of OSA-related intermittent hypoxia or rather a reflection of underlying obesity and a chronic inflammatory state.

Accordingly, given that OSA induces recurrent hypoxia and an inflammatory response, elucidating the relationship between hepcidin and these processes is of particular importance. The primary aim of this study was to evaluate the association between serum hepcidin levels and the presence and severity of OSA. The secondary aim was to investigate whether this observed association was independent of obesity and C-reactive protein levels, a marker of systemic inflammation. In this context, it was hypothesized that hepcidin may serve as a potential biomarker reflecting the hypoxic and inflammatory pathophysiological mechanisms specific to OSA.

## 2. Materials and Methods

### 2.1. Study Design and Patients

A total of 136 individuals who presented to the chest diseases outpatient clinic with complaints of snoring, excessive daytime sleepiness, and nocturnal sleep apnea between April 2025 and December 2025 and underwent polysomnography (PSG) with a preliminary diagnosis of OSA were included in the study.

The study included cases aged 18 years and older who underwent a full-night PSG. Individuals with acute infection, chronic inflammatory disease, cancer, rheumatological disease, blood transfusion within the last 3–6 months, use of iron metabolism-related medication, liver or kidney failure, and cases with sleep efficiency below 60% or total sleep time below 180 min in PSG results were excluded from the study. Additionally, participants underwent a detailed clinical interview and physical examination to screen for other primary sleep disorders such as restless legs syndrome, insomnia, and narcolepsy, and those with such conditions were excluded to ensure the integrity of the comparison groups.

OSA severity was classified based on AHI; individuals with AHI < 5 were included in the control group, those with 5–15 in the mild group, those with 15–30 in the moderate group, and those with ≥30 in the severe OSA group. The control group consisted of individuals who were confirmed to be otherwise healthy and free of any sleep-disordered breathing.

Participants’ anthropometric measurements were performed using standard methods; body weight and height were measured, and Body Mass Index (BMI) was calculated. Neck circumference was measured at the level of the cricothyroid membrane while standing. To evaluate clinical and anatomical risk factors for OSA, validated screening tools and scoring systems were applied. The Epworth Sleepiness Scale (ESS), a validated self-administered questionnaire consisting of eight items, was used to quantify subjective daytime sleepiness. Scores ranging from 0 to 24 were recorded, with values greater than 10 indicating excessive daytime sleepiness. In addition, the STOP-BANG questionnaire—an eight-item screening tool comprising Snoring, Tiredness, observed apnea, high blood Pressure, BMI, Age, Neck circumference, and Gender—was employed to determine the clinical probability of OSA. The Mallampati score was used to assess upper airway anatomy by classifying the visibility of oropharyngeal structures, thereby providing a standardized indicator of potential airway crowding. All questionnaires and clinical assessments were administered by trained physicians before polysomnography to ensure standardized data collection.

The study was conducted in accordance with the Declaration of Helsinki and was approved by the Ethics Committee of Balıkesir University Faculty of Medicine (Date: 11 March 2025, decision number: 2025/140). Written informed consent was obtained from all individuals participating in the study.

### 2.2. Polysomnography Evaluations

All patients underwent overnight PSG using a 62-channel Embla N7000 device (Medcare Flage, Reykjavik, Iceland) in a laboratory setting. The examinations were performed in accordance with the current criteria of the American Academy of Sleep Medicine (AASM). The recordings simultaneously monitored electroencephalogram (EEG) (C3-A2, C4-A1, O1-A2, F4-A1), electrooculogram (EOG), electrocardiogram (ECG), submental and tibialis anterior electromyogram (EMG), oro-nasal airflow, thoracic and abdominal movements, and oxygen saturation. Sleep stages were manually scored in 30-s epochs; total sleep time, sleep efficiency, sleep architecture, apnea- hypopnea index (AHI), oxygen desaturation index (ODI), minimum oxygen saturation, and arousal index were analyzed.

ODI was defined as the average number of oxygen desaturation episodes per hour of sleep. A desaturation episode was identified as a decrease in mean oxygen saturation of ≥3% from the baseline, lasting for at least 10 s. ODI values were analyzed as a continuous variable representing the frequency of oxygen desaturation events per hour of sleep. All recordings were obtained by experienced and certified technicians and verified by a pulmonologist.

### 2.3. Laboratory Assessments

Morning venous blood samples were collected between 08:00 and 09:00 a.m. after the overnight sleep test, following an 8–12-h fast. Serum samples were allowed to clot in biochemistry tubes, then centrifuged at 4000 rpm for 10 min at 4 °C. A portion of the serum was placed in Eppendorf tubes for hepcidin analysis and stored at −40 °C.

Complete blood counts were performed using a DxH 800 hematology analyzer (Beckman Coulter, Brea, CA, USA). Serum iron and unsaturated iron-binding capacity (UIBC) were measured using an AU680 analyzer (Beckman Coulter Inc., Brea, CA, USA), and ferritin levels were measured using a UniCel DxI 600 analyzer (Beckman Coulter, Brea, CA, USA). Serum C-reactive protein (CRP) levels were determined using the BN II analyzer (Siemens Healthineers, Marburg, Germany). Transferrin saturation percentage, TSAT (%) = [iron (µmol/L)/TDBK (µmol/L)] × 100, was calculated using the formula. All analyses were performed at the Biochemistry and Microbiology Laboratories of Balıkesir University Health Application and Research Hospital.

### 2.4. Serum Hepcidin Analysis

Serum hepcidin levels were determined using a commercially available enzyme-linked immunosorbent assay (ELISA) kit (Human Hepcidin ELISA, Elabscience, Catalog No: E-EL-H6202, Houston, TX, USA). Measurements were performed spectrophotometrically using a multimode microplate reader (Varioscan Flash Multimode Reader, Thermo Scientific, Waltham, MA, USA) operating at a wavelength of 450 nm.

The measurement range of the kits used is 0.78–50 ng/mL, with a sensitivity of 0.32 ng/mL and a coefficient of variation below 10%. All analyses were performed according to the manufacturer’s instructions. Serum hepcidin measurements were performed at the Medical Biochemistry Laboratory of Balıkesir University Faculty of Medicine.

### 2.5. Statistical Analysis

Statistical analyses were performed using IBM SPSS 25.0 software (SPSS Inc., Chicago, IL, USA). The distribution characteristics of continuous variables were evaluated using the Kolmogorov–Smirnov test. Data showing a normal distribution are presented as the mean ± standard deviation, and data not showing a normal distribution are presented as the median (interquartile range). For comparisons between two groups, the independent samples *t*-test was used when the assumption of normal distribution was met, and the Mann–Whitney U test was used when it was not met. For comparisons of three or more groups, one-way analysis of variance (ANOVA) was applied for normally distributed variables, and the Kruskal–Wallis test was applied for non-normally distributed variables. Appropriate post hoc analyses were used for pairwise comparisons after ANOVA.

The chi-square test was applied for comparisons of categorical variables. The relationships between hepcidin levels and clinical, polysomnographic, and laboratory parameters were evaluated using Pearson or Spearman correlation analyses, depending on the distribution characteristics of the variables. Linear regression analyses were performed to determine the independent factors affecting hepcidin levels; univariate analyses were first applied, followed by the creation of a multiple linear regression model to assess the effect of potential confounders. Body mass index (BMI) was included as a control variable in this model and the interaction between apnea–hypopnea index (AHI) and BMI (AHI × BMI) was also tested. Model fit was assessed using the coefficient of determination (R^2^). In all statistical analyses, *p* < 0.05 was considered statistically significant.

## 3. Results

### Findings

The demographic and clinical characteristics of the patient groups are presented in Table 1. A total of 136 cases were included in the study, 34 of whom were in the control group (AHI < 5) and 102 of whom had a diagnosis of obstructive sleep apnea (OSA; AHI ≥ 5). The mean age of the OSA group was significantly higher than that of the control group (*p* = 0.003). No significant difference was found between the groups in terms of gender distribution (*p* = 0.23) (Table 1).

BMI and neck circumference were significantly higher in the OSA group compared to the control group (*p* < 0.001 and *p* < 0.001, respectively). The Mallampati score, STOP-BANG score, and Epworth Sleepiness Scale scores were also significantly higher in the OSA group (*p* < 0.001, *p* < 0.001, and *p* < 0.001, respectively). Consistent with the literature, AHI and oxygen desaturation index were significantly higher in the OSA group compared to the control group (*p* < 0.001 and *p* < 0.001, respectively) (Table 1).

When polysomnographic parameters were evaluated, mean oxygen saturation and minimum oxygen saturation values were found to be significantly lower in the OSA group compared to the control group (*p* < 0.001 and *p* < 0.001, respectively) (Table 1).

No statistically significant differences were observed between the groups in terms of iron, ferritin, hemoglobin, transferrin saturation, and UIBC levels (*p* > 0.05). However, erythrocyte sedimentation rate was significantly higher in the OSA group (*p* = 0.02). Although C-reactive protein levels were higher in the OSA group, this difference did not reach statistical significance (*p* = 0.06) (Table 1).

Hepcidin levels were significantly higher in the OSA group compared to the control group (*p* < 0.001).

OSA severity classification in this study was based on the Apnea–Hypopnea Index (AHI). OSA patients included in the study were divided into three groups according to disease severity: mild (n = 26), moderate (n = 34), and severe (n = 42) (Table 2). No significant difference was found between the groups in terms of mean age and gender distribution (*p* = 0.754 and *p* = 0.908, respectively) (Table 2).

Body mass index increased significantly as OSA severity increased (*p* = 0.005). Similarly, neck circumference measurement increased in parallel with OSA severity (*p* = 0.0034). In the upper airway assessment, the Mallampati score was significantly different between OSA groups (*p* < 0.001).

When clinical screening and symptom scores were examined, STOP-BANG and Epworth Sleepiness Scale scores increased significantly as OSA severity increased (*p* = 0.005 and *p* < 0.001, respectively). When polysomnographic parameters were evaluated, AHI and ODI values showed significant differences between groups, with the highest values found in the severe OSA group (*p* < 0.001 and *p* < 0.001, respectively).

Regarding nocturnal oxygenation parameters, mean oxygen saturation decreased as OSA severity increased (*p* = 0.011), while the lowest oxygen saturation showed a significant difference between groups and was significantly lower in the severe OSA group (*p* < 0.001).

When iron metabolism parameters were evaluated, no statistically significant difference was found between the groups in terms of serum iron, transferrin saturation, UIBC, and ferritin levels. Hemoglobin levels were also similar according to OSA severity (*p* = 0.664) (Table 2).

When inflammatory markers were examined, C-reactive protein (CRP) levels showed a significant difference between groups and were found to be higher in the severe OSA group (*p* = 0.017), while no significant difference was observed between groups in terms of sedimentation rate (*p* = 0.28).

**Figure 1:**
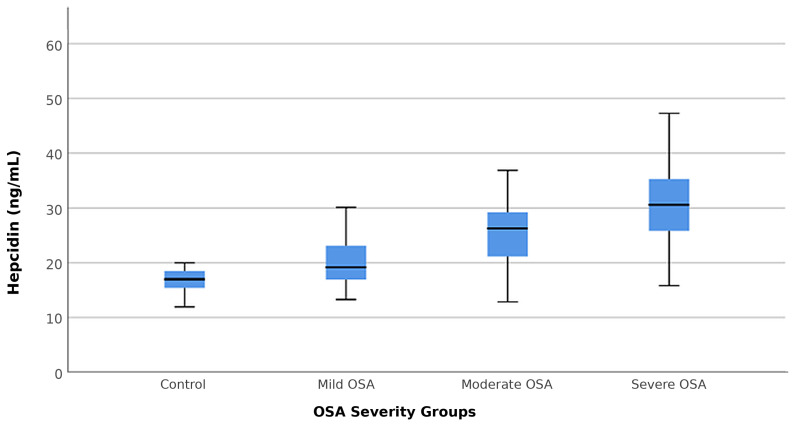
Distribution of hepcidin levels according to OSA groups.

Hepcidin levels showed a significant difference according to OSA severity, and mean hepcidin values showed a gradual increase from the mild OSA group to the severe OSA group (*p* < 0.001) (Figure 1).

The relationships between hepcidin levels and clinical, polysomnographic, and laboratory parameters were evaluated (Table 3). BMI and neck circumference showed a significant positive correlation with hepcidin (r = 0.316 and r = 0.344, respectively; *p* < 0.001, *p* < 0.001). A positive and significant correlation was found between STOP-BANG and Epworth Sleepiness Scale scores and hepcidin (r = 0.482 and r = 0.516, respectively; *p* < 0.001, *p* < 0.001) (Table 3).

In terms of polysomnographic parameters, a positive correlation was found between AHI and ODI and hepcidin (r = 0.678 and r = 0.655, respectively; *p* < 0.001, *p* < 0.001). When nighttime oxygenation parameters were evaluated, a significant negative correlation was observed between mean oxygen saturation and minimum oxygen saturation and hepcidin levels (r = −0.339 and r = −0.531, respectively; *p* < 0.001, *p* < 0.001) (Table 3).

When parameters related to iron metabolism were examined, no statistically significant correlation was found between transferrin saturation, serum iron, UIBC, ferritin levels, and hepcidin. Similarly, no significant relationship was observed between hemoglobin level and hepcidin (*p* = 0.19). Among inflammatory markers, a weak but statistically significant positive correlation was found between CRP levels and hepcidin (r = 0.227; *p* = 0.008), while no significant relationship was observed between sedimentation rate and hepcidin (*p* = 0.51) (Table 3).

**Table 3:** Relationship between hepcidin levels and clinical, polysomnographic, and laboratory parameters.

Hepcidin	r	*p*-Value
BMI (kg/m^2^)	0.316	<0.001 **
Neck circumference	0.344	<0.001 **
STOP-BANG	0.482	<0.001 **
Epworth	0.516	<0.001 *
AHI	0.678	<0.001 *
ODI	0.655	<0.001 *
Average oxygen Saturation (%)	−0.339	<0.001 **
Lowest oxygen Saturation (%)	−0.531	<0.001 **
Serum transferrin (g/L)	−0.136	0.136 *
Iron (µg/dL)	−0.058	0.513 **
UIBC (µg/dL)	0.130	0.134 *
Ferritin (µg/L)	0.022	0.806 **
Hemoglobin (g/dL)	0.113	0.190 **
C-reactive protein (mg/L)	0.227	0.008 **
Sedimentation rate (mm/hour)	0.057	0.510 *

BMI: body mass index; ODI: oxygen saturation index; AHI: apnea-hypopnea index; UIBC: unsaturated iron-binding capacity. ** Spearman correlation test, * Pearson correlation test, r: correlation coefficient. The relationships between serum hepcidin levels and OSA-related parameters were evaluated using univariate and multiple linear regression analysis (Table 4). According to univariate analysis results, serum hepcidin levels were significantly associated with AHI (B = 0.211; 95% CI: 0.172–0.250; *p* < 0.001), BMI (B = 0.496; 95% CI: 0.268–0.724; *p* < 0.001), and C-reactive protein (B = 0.539; 95% CI: 0.072–1.006; *p* = 0.024). In the multivariable linear regression model controlling for potential confounders, only AHI continued to have an independent and significant association with serum hepcidin levels (B = 0.257; 95% CI: 0.066–0.448; *p* < 0.001). In this model, the associations with BMI and CRP and the AHI × MI interaction were not statistically significant (BMI: B = 0.079, *p* = 0.593; CRP: B = −0.023, *p* = 0.906; AHI × BMI interaction: B = −0.001, *p* = 0.605).

**Table 4:** Unadjusted and multivariable-adjusted associations between hepcidin levels and OSA-related parameters.

Variable	Unadjusted B (95%CI)	*p*-Value	Adjusted B (95% CI)	*p*-Value
AHI	0.211 (0.172–0.250)	<0.001	0.257 (0.066–0.448)	<0.001
BMI kg/m^2^	0.496 (0.268–0.724)	<0.001	0.079 (−0.214–0.372)	0.593
C-reactive protein (mg/L) AHI × BMI interaction	0.539 (0.072–1.006)	0.024	−0.023 (−0.406–0.360) −0.001 (−0.007–0.004)	0.906 0.605

BMI: body mass index kg/m^2^, AHI: apnea-hypopnea index (/hour), B: Unstandardized coefficient; CI: Confidence interval. The multivariable model explained 46.8% of the variance in serum hepcidin levels (R^2^ = 0.468, adjusted R^2^ = 0.452).

## 4. Discussion

Our study demonstrated that serum hepcidin levels were significantly higher in OSA patients compared to the control group. The gradual increase in hepcidin levels observed as AHI increased suggests a strong and linear relationship between the two variables. Although hepcidin elevation secondary to chronic inflammation in obesity has been previously reported, the fact that the AHI–hepcidin relationship remained significant after controlling for the effects of BMI and CRP in the multivariate regression analysis suggests that hepcidin elevation is associated with pathophysiological processes specific to OSA.

OSA constitutes a significant morbidity burden due to its strong association with cardiovascular and metabolic diseases [10]. Therefore, there remains a need to identify sensitive and specific biomarkers that can simultaneously reflect the systemic inflammation and intermittent hypoxia accompanying the disease.

Hepcidin is a central regulator that maintains the balance between inflammation, iron load, and erythropoietic requirements, and is considered one of the primary mediators of changes in iron metabolism, particularly in chronic inflammatory conditions [7]. Studies have reported that oxidative stress and systemic inflammation in OSA lead to functional iron deficiency by disrupting iron metabolism [11].

It has been suggested that increased erythropoietic stimulation accompanying intermittent hypoxia may exert a counter-regulatory effect on hepcidin synthesis via the erythropoietin–erythroferron axis [12]. This suggests that hepcidin regulation in OSA is shaped not by a unidirectional mechanism, but by the dynamic interaction between inflammation and erythropoietic activity. In our study, the parallel increase in hepcidin levels with disease severity and hypoxic load suggests that the inflammatory stimulus overrides erythropoietic inhibitory signals and that the net biological effect is toward increased hepcidin. Although there are a limited number of studies on this topic in the literature, one study found that hepcidin levels were significantly increased in OSA patients [13]; another study showed that hepcidin levels were associated with both the presence of OSA and disease severity [14].

There is no consensus in the literature regarding the effect of hypoxia on hepcidin regulation; existing data point to different and sometimes contradictory mechanisms. Some experimental studies have shown that hypoxia stimulates erythropoiesis by increasing erythropoietin release via HIF-1α-mediated pathways, and that hepcidin synthesis is secondarily suppressed in this process [14]. In contrast, studies conducted using a different approach have reported that hypoxia can directly increase hepcidin expression at the hepatocyte level, particularly through oxidative stress. Indeed, Cui and colleagues demonstrated that hypoxia-induced hepcidin increase in vivo models is associated with the activation of the IL6/JAK2/STAT3 signaling pathway [9].

Data obtained at the clinical level also support this complex regulation. Hikmet Fırat et al. detected a significant increase in hepcidin levels throughout the night in individuals with OSA and associated this increase with the inflammatory response triggered by repeated episodes of hypoxia–reoxygenation [15]. The strong ODI–hepcidin relationship observed in our study suggests that hepcidin may be a biomarker reflecting not only the inflammatory burden but also the severity of intermittent hypoxia, which is a fundamental pathophysiological feature of OSA.

The findings are consistent with previous data suggesting that hepcidin may be part of an inflammation-related response in respiratory system diseases. It has been reported that hepcidin plays a role in alveolar macrophage function in chronic obstructive pulmonary disease models, while serum hepcidin levels are elevated in patients with idiopathic pulmonary fibrosis and that alveolar epithelial cells contribute to this increase [16,17]. This suggests that hepcidin is associated not only with systemic but also with inflammatory and structural processes occurring at the pulmonary level.

The significant relationship between Mallampati stage and hepcidin levels suggests that increased collapse tendency in the upper airway may lead to more intense obstruction episodes and associated increased hypoxia, thereby affecting the levels of this molecule. Similarly, the positive correlation between the Epworth sleepiness scale and hepcidin can be considered a biochemical reflection of the interaction between excessive daytime sleepiness and systemic stress and inflammation.

The relationship between hepcidin and obesity has been clearly demonstrated in previous studies; it is known that obesity, as a chronic and low-grade inflammatory condition, can increase hepcidin synthesis [18]. Consistent with the literature, our study found that traditional markers of systemic inflammation, such as C-reactive protein (CRP) and erythrocyte sedimentation rate (ESR), were positively correlated with OSA severity (AHI). This suggests that as the severity of OSA increases, the systemic inflammatory response also intensifies. The literature reports conflicting findings regarding the relationship between inflammation markers and hepcidin; while some studies have found positive relationships, others have not detected any significant relationship [19,20]. However, the most notable finding of our study is that while CRP and ESR showed significant associations with hepcidin in univariate analyses, only AHI remained a strong independent predictor of serum hepcidin levels in the multiple regression model, independent of these inflammatory markers. In our study, although significant relationships were observed with BMI and CRP in univariate analysis, the fact that only AHI remained significant as an independent predictor of serum hepcidin levels in the multiple regression model indicates that this relationship is independent of obesity and general inflammation. Moreover, the AHI × BMI interaction term was not significant, suggesting that the effect of OSA severity on hepcidin is not modified by obesity. This finding suggests that hepcidin may be directly related to the hypoxic and inflammatory processes specific to OSA.

Limitations of this study include its single-center design, limited sample size, which may restrict the generalizability of the findings, and the fact that polysomnographic assessments were based on single-night recordings. Therefore, further prospective, multicenter studies with larger patient populations are needed to validate these results.

## 5. Conclusions

Our study demonstrates that elevated serum hepcidin levels in OSA patients are directly associated with disease severity, independent of obesity and general inflammation markers. This finding suggests that increased hepcidin may reflect pathophysiological processes associated with the recurrent hypoxic stress specific to OSA. In conclusion, the data obtained indicate that hepcidin may contribute to clinical research as a potential biomarker for assessing OSA severity.

## Figures and Tables

**Table 1:** Comparison of demographic, clinical, polysomnographic, and laboratory characteristics of the study groups.

	Control (AHI < 5) n: 34	OSA (AHI ≥ 5) n: 102	*p*-Value
Age	41.53 ± 12.43	48.83 ± 11.92	0.003
Female/Male, n (%)	13/21 (38.2/61.8)	28/74 (27.5/72.5)	0.235
BMI (kg/m^2^)	28.87 ± 4.01	33.02 ± 6.01	<0.001
Neck Circumference (cm)	39 ± 2.93	41.52 ± 3.41	<0.001
Mallampati	1.38 ± 0.73	2.79 ± 0.90	<0.001
STOP-BANG	3.61 ± 1.25	5.07 ± 1.23	<0.001
Epworth	4.79 ± 3.79	10.86 ± 6.19	<0.001
AHI	2.63 ± 1.42	33.52 ± 26.84	<0.001
ODI	2.44 ± 1.46	31.78 ± 26.85	<0.001
Average oxygen saturation (%)	94.10 (89.7–97.6)	92.70 (80.8–96.8)	<0.001
Lowest oxygen Saturation (%)	90.0 (81–94)	83.0 (50–90)	<0.001
Iron (µg/dL)	76.0 (23–148)	71.0 (15–379)	0.231
Serum transferrin (g/L)	25.73 ± 12.62	22.70 ± 10.76	0.242
UIBC (μg/dL)	272 ± 70.15	276.42 ± 82.95	0.773
Ferritin (µg/L)	37.5 (4.4–101.9)	32.1 (5.9–364)	0.553
Hemoglobin (g/dL)	14.3 (10.8–16.7)	14.6 (12.0–39.5)	0.218
C-reactive protein (mg/L)	3.0 (3.0–9.37)	3.0 (3.0–19.6)	0.068
Sedimentation rate	12.35 ± 7.93	16.39 ± 10.76	0.022
Hepcidin (ng/mL)	16.61 ± 2.28	26.82 ± 8.05	<0.001

BMI: body mass index; ODI: oxygen desaturation index; AHI: apnea-hypopnea index, UIBC: unsaturated iron-binding capacity. Continuous variables are presented as the mean ± standard deviation or the median (minimum–maximum) according to their distribution characteristics. The level of statistical significance was set at *p* < 0.05.

**Table 2:** Demographic, clinical, polysomnographic, and laboratory findings of the study population according to OSA severity.

	Mild OSA n: 26	Moderate OSA n: 34	Severe OSA n: 42	*p*-Value
Age	47.42 ± 11.07	49.76 ± 11.04	48.95 ± 13.24	0.754
Female/Male, n (%)	8/18 (28.6/24.3)	9/25 (32.1/33.8)	11/31 (39.3/41.9)	0.908
BMI (kg/m^2^)	30.94 ± 5.27	31.85 ± 4.71	35.25 ± 6.72	0.005
Neck Circumference (cm)	40.73 ± 2.61	40.85 ± 3.08	42.57 ± 3.87	0.034
Mallampati	1.92 ± 0.39	2.64 ± 0.73	3.45 ± 0.73	<0.001
STOP-BANG	4.53 ± 0.94	4.97 ± 1.02	5.50 ± 1.40	0.005
Epworth	6.69 ± 5.48	10.85 ± 5.69	13.45 ± 5.67	<0.001
AHI	9.19 ± 2.72	19.53 ± 4.03	59.90 ± 22.53	<0.001
ODI	7.90 ± 2.99	18.52 ± 3.84	57.28 ± 24.19	<0.001
Average oxygen Saturation (%)	93.2 (85.5–96.8)	92.6 (80–96.3)	91.9 (80.9–95.5)	0.011
Lowest oxygen Saturation (%)	87.0 (69–90)	84.0 (69–90)	78.0 (50–87)	<0.001
Iron (µg/dL)	76.5 (27–352)	76.0 (15–195)	68.0 (17–379)	0.931
Serum transferrin (g/L)	24.08 ± 11.09	23.06 ± 11.78	21.55 ± 9.08	0.633
UIBC (µg/dL)	263.12 ± 78.55	285.78 ± 68.89	277.31 ± 95.86	0.583
Ferritin (µg/L)	33.0 (6.8–178)	39.6 (5.9–364)	31.4 (6.40–325)	0.762
Hemoglobin (g/dL)	14.35 (12.30–38.40)	14.30 (12–17.7)	14.90 (12.1–39.5)	0.664
C-reactive protein (mg/L)	3.0 (3–14)	3.0 (3–8)	4.06 (3–19.6)	0.017
Sedimentation rate (mm/hour)	15.76 ± 9.80	14.47 ± 10.32	18.33 ± 11.58	0.284
Hepcidin (ng/mL)	20.23 ± 4.46	25.81 ± 5.27	31.71 ± 8.50	<0.001

BMI: body mass index; ODI: oxygen saturation index; AHI: apnea-hypopnea index; UIBC: unsaturated iron-binding capacity. Data are expressed as the mean ± standard deviation or the median (minimum–maximum) where appropriate. One-way ANOVA or Kruskal–Wallis test was applied for continuous variables, and chi-square test for categorical variables. *p* < 0.05 was considered statistically significant.

## Data Availability

The data can be obtained from the corresponding author upon reasonable request.
